# Increased cerebrovascular reactivity in selected brain regions after extracranial-intracranial bypass improves the speed and accuracy of visual cancellation in patients with severe steno-occlusive disease: a preliminary study

**DOI:** 10.1007/s10143-021-01720-0

**Published:** 2022-01-30

**Authors:** Koji Shimonaga, Seiji Hama, Akira Furui, Akiko Yanagawa, Akihiko Kandori, Hirokazu Atsumori, Shigeto Yamawaki, Toshinori Matsushige, Toshio Tsuji

**Affiliations:** 1 Department of Neurosurgery and Interventional Neuroradiology, Hiroshima City Asa Citizens Hospital, Hiroshima, 731-0293 Japan; 2 Department of Neurosurgery, Graduate School of Biomedical and Health Sciences, Hiroshima University, 1-2-3 Kasumi, Minami-ku, Hiroshima, 734 8551 Japan; 3 Department of Rehabilitation, Hibino Hospital, Hiroshima, 731-3164 Japan; 4 Center for Brain, Mind and KANSEI Sciences Research, Hiroshima University, Hiroshima, 734‑8551 Japan; 5 Graduate School of Advanced Science and Engineering, Hiroshima University, Hiroshima, 739-8527 Japan; 6 Center for Exploratory Research, Research and Development Group, Hitachi. Ltd, Tokyo, 185-8601 Japan

**Keywords:** Extracranial-intracranial bypass, Cognitive function, Speed-accuracy trade-off, Severe steno-occlusive disease, Visual cancellation

## Abstract

The effect of the change in cerebrovascular reactivity (CVR) in each brain area on cognitive function after extracranial-intracranial bypass (EC-IC bypass) was examined. Eighteen patients who underwent EC-IC bypass for severe unilateral steno-occlusive disease were included. Single-photon emission CT (SPECT) for evaluating CVR and the visual cancellation (VC) task were performed before and after surgery. The accuracy of VC was expressed by the arithmetic mean of the age-matched correct answer rate and the accurate answer rate, and the averages of the time (time score) and accuracy (accuracy score) of the four VC subtests were used. The speed of VC tended to be slower, whereas accuracy was maintained before surgery. The EC-IC bypass improved CVR mainly in the cerebral hemisphere on the surgical side. On bivariate analysis, when CVR increased post-operatively, accuracy improved on both surgical sides, but the time score was faster on the left and slower on the right surgical side. Stepwise multiple regression analysis showed that the number of the brain regions associated with the time score was 5 and that associated with the accuracy score was 4. In the hemodynamically ischemic brain, processing speed might be adjusted so that accuracy would be maintained based on the speed-accuracy trade-off mechanism that may become engaged separately in the left and right cerebral hemispheres when performing VC. When considering the treatment for hemodynamic ischemia, the relationship between CVR change and the speed-accuracy trade-off in each brain region should be considered.

**Supplementary Information:**

The online version contains supplementary material available at 10.1007/s10143-021-01720-0.

## Introduction

Symptomatic major cerebral arterial occlusion or stenosis makes the status of cerebral hemodynamics progress from stage 0 (normal cerebral hemodynamics) to stage 1 hemodynamic ischemia (stage 1) (autoregulatory vasodilation to keep cerebral blood flow (CBF) normal) and then stage 2 hemodynamic ischemia (stage 2) (CBF cannot be maintained, and brain tissues increase the oxygen extraction fraction (OEF)) as the stenosis progresses, and cerebral infarction becomes inevitable before long [1–4]. To prevent progression from stage 2 to cerebral infarction, extracranial-intracranial (EC-IC) bypass surgery has been considered [5–7]. However, the selection criteria for medical treatment or surgical treatment have been controversial until now [4, 8]. One of the key issues lies in how to evaluate stage 2, especially the signs of progression to cerebral infarction.

Although positron emission tomography (PET) has the advantage of being able to diagnose stage 2 because OEF can be measured [4], its clinical availability is limited by its high cost and complexity [2]. Instead, single-photon emission CT (SPECT)–measured cerebrovascular reactivity (CVR), which is defined as the change in CBF in response to a vasoactive stimulus, has been used [2, 3, 9]. According to a Japanese Extracranial-Intracranial Bypass Trial (JET) study, if rest CBF was less than 80% of the normal value and CVR was less than 10%, EC-IC bypass surgery was more effective in preventing cerebral infarction than drug therapy [5, 6].

Cerebral hypoperfusion induced by steno-occlusive diseases has been thought to be associated with cognitive decline [10–12]. If so, the status of cerebral hemodynamics (especially stage 2) may be estimated using cognitive function tests. Some previous reports suggested that EC-IC bypass improves cognitive function in patients with steno-occlusive disease [11, 12]. However, these previous studies have some limitations, and the effect of EC-IC bypass on cognitive function has remained controversial [12, 13].

CVR shows the ability of the cerebral vascular bed (regulation of vasodilatation and collateral blood flow) to maintain a balanced distribution of blood flow and varies by brain region [3]. In addition, cognitive function is classified into many types, each of which is associated with a specific brain region [14, 15]. Thus, the association between CVR and cognitive function before and after EC-IC bypass should be examined after dividing the brain into regions.

We previously demonstrated that right hemisphere damage led to a delay in cognitive processing speed after stroke [16]. Skurvydas and colleagues studied hand and leg movement control in stroke patients in the right cerebral hemisphere and showed that they perform impulsive movements at the expense of inaccurate movements [17]. This is a well-known theory in the kinesiology or neuroscience, but it is little known in the stroke or neurosurgical field, called the “speed-accuracy trade-off,” which implies that faster movement can be performed at the cost of reduced accuracy and vice versa [18]. The speed-accuracy trade-off mechanism might be associated with cognitive function in the process from hemodynamic ischemia (especially stage 2) to cerebral infarction.

The present study was a preliminary test of the hypothesis that “regional CVR changes” and “speed-accuracy trade-off theory” can be used to clarify the relationship between cognitive function and CVR, to clarify the cognitive dysfunction that accompanies the progression from hemodynamic ischemia to cerebral infarction.

## Material and methods

### Patients

This retrospective study was conducted as a preliminary study after approval by the Research Ethics Committee of Hiroshima City Asa Citizens Hospital and Hiroshima University (E-466–3, E-1554–2). Written, informed consent was obtained from all participants. In this study, 18 consecutive patients who underwent EC-IC bypass for severe unilateral steno-occlusive disease of the intracranial internal carotid artery (ICA) or middle cerebral artery (MCA) at Asa Citizens Hospital from September 2017 to April 2020 were reviewed. All patients experienced transient ischemic attacks (TIAs) or non-disabling strokes within 6 months derived from the hemisphere ipsilateral to the lesion.

CBF and CVR were estimated by SPECT. The regional CBF (rCBF) of the ipsilateral cerebral cortex region was compared by the value divided by the rCBF of the ipsilateral cerebellar cortex region [19]. Then, according to the JET study, EC-IC bypass was indicated for rCBF reduction of less than 80% and regional CVR (rCVR) reduction of less than 10% [5, 6]. In all cases, no stenotic lesions causing decreased cerebellar blood flow were observed in the vertebrobasilar arteries.

The inclusion criteria for this study were patients with available pre-operative and post-operative (3–6 months or later after surgery) CVR studies. Exclusion criteria were as follows: (1) allergy to contrast media; (2) renal dysfunction (estimated glomerular filtration rate < 30 ml/min/1.73 m^2^); or (3) medical illness, physical disability, or aphasia (modified Rankin scale score ≥ 3) precluding the VC task. Eighteen patients were selected on the basis of the inclusion criteria (5 women; mean age at the time of bypass 68.1 years). The etiology included atherosclerosis (*n* = 18) (Table 1).

**Table 1:** Patients’ demographics

	All	Side of EC-IC bypass		
		Lt (*n* = 11)	Rt (*n* = 7)	*p*
Age ± SE (y)	68.111 ± 9.934	67.364±10.043	69.286±10.436	0.751
Sex; male (%)	13 (72.22%)	8 (72.73%)	5 (71.43%)	0.952
Time score				
Pre-op ± SE	1.801 ± 1.202	1.675 ± 0.643	2.001 ± 1.825	0.684
Post-op ± SE	1.684 ± 1.427	1.502 ± 0.575	1.970 ± 2.250	0.497
Change ± SE	− 0.117 ± 0.436	− 0.172 ± 0.383	− 0.031 ± 0.530	0.964
Accuracy score				
Pre-op ± SE	0.987 ± 0.027	0.982 ± 0.0280	0.994 ± 0.024	0.16
Post-op ± SE	0.991 ± 0.014	0.988 ± 0.016	0.996 ± 0.009	0.222
Change ± SE	0.005 ± 0.025	0.006 ± 0.029	0.002 ± 0.016	0.751

The Mann–Whitney test was used to compare continuous variables, and the *χ*^2^ test was used to compare categorical variables

*SE* standard error

### Assessment of cognitive functions (time and accuracy score)

We routinely perform cognitive function tests (mini-mental state examination, trail making test, and the Clinical Assessment for Attention (CAT)) within 3 days of SPECT before and 3–6 months after surgery. Of these cognitive tests, the duration and accuracy of visual cancellation (VC), position Stroop test, and Continuous Performance Test could be evaluated at the same time, but only the VC was performed in all 18 cases. Thus, the results of VC were used for the following analysis. VC consisted of four kinds of subtests (Kana, Triangle, Symbol, Number) included in CAT, which is a standardized test for attention deficit, that were used as previously described [15]. Participants used a pencil to cross out a target stimulus dispersed within rows of randomly placed interfering stimuli displayed on a sheet. These tasks were scored as speed (completion time) and accuracy. Accuracy was based on the ratio of the number of correct answers to the total number of items (% correct answers) or the number of accurate answers compared to the number of total responses (both correct and incorrect responses) (% accurate answer).

It is known that the scores of the VC depend on age. To correct by age, age-matched values were calculated as (VC score) / (age-specific mean VC value); the lower the age matched % correct answer and % accurate answer, the greater the attentional disturbance, and the higher the age-matched completion time (the slower the speed), the greater the attentional disturbance.

The accuracy of each VC subtest was evaluated by (age matched % correct answer + age-matched % accurate answer) / 2, and the following analysis was performed with the average value of the four accuracies (accuracy score). In addition, the following analysis was performed using the average value of the age-matched completion times of four VCs (time score). A score of 1 of both the accuracy score and the time score indicates the age-corrected average value.

### Measurement and analysis of CBF and CVR using SPECT

All subjects in this study received a 222-MBq dose of *N*-isopropyl-^123^I-*p*-iodoamphetamine (IMP) intravenously. Siemens e.cam (Siemens Medical Solutions, Erlangen, Germany) was used in 18 patients to acquire the projection data in a continuous mode at 150 s/cycle (180° rotation of dual heads) for 2 cycles, repeated 6 times. In both machines, low-energy high-resolution collimators were used, with a matrix size of 128 × 128 and an energy window of 159 keV ± 15%. After the data were obtained, a three-dimensional stereotactic surface profile program (3D-SSP, Nihon Medi-Physics, Tokyo, Japan) was used to spatially normalize the local distribution. In brief, the coordinate data were converted to the normal brain based on the Talairach brain atlas, classified into segments, and the Z score was calculated by comparing with a normal database using SEE (stereotactic extraction estimation) analysis, and the CBF and CVR in each brain region were evaluated [20].

In this study, the rCBF values were obtained by SPECT with the graph plot method that uses IMP, which does not require arterial blood sampling [21]. Thus, arterial sampling was not performed during SPECT.

Scans for rCBF were performed just before and 10 min after injection of 1.0 g of acetazolamide. Regional cerebrovascular reactivity (rCVR) was calculated as follows: rCVR(%) = [(acetazolamide challenge rCBF − resting rCBF) / resting rCBF] × 100. The change in CVR (post-operative CVR − pre-operative CVR) was calculated.

The CVR was divided into three areas (ACA, MCA, and PCA) or the 31 supratentorial areas of 50 regions of level 3 of the anatomical classification based on the Talairach Daemon database on each side (supplemental Table 1) [20].

### Operative procedure

Under general anesthesia, with continued antiplatelet medication perioperatively, a skin incision was made just over the superficial temporal artery (STA) frontal branch or parietal branch. Under microscopy, meticulous STA dissection was conducted. The skin incision was then extended toward the forehead, and a skin flap was reflected. The frontal branch of the STA was dissected. After craniotomy, an STA-MCA single or double anastomosis was performed between each STA branch and the recipient M4 (cortical MCA branch). Successful bypass was confirmed by microvascular Doppler evaluation. EC-IC bypass in this study included only the direct procedure and was not combined with indirect bypass procedures.

### Statistical analysis

To compare differences between two groups, Fisher’s exact test was used for categorical variables, and the Mann–Whitney *U*-test was used for quantitative variables. The level of significance was set at *p* < 0.05.

To test the correlations between the time or the accuracy score and the CVR in the 31 brain areas on each side (62 in total), bivariate analysis (Spearman’s rank correlation coefficient, *ρ*) was performed.

Stepwise multiple linear regression analysis was used to estimate the independent effects of predictor variables on the time or accuracy score (forward–backward selection method). These predictor variables were as follows: age, sex, laterality of the operation, and the CVR changes in the brain region with a *p*-value of 0.05 or less in the above bivariate analysis. Logworth was calculated as -log_10_ (*p* value), and higher values were more significant. To assess multicollinearity, the variance inflation factor (VIF) was calculated. A value of 10 was considered to be sufficiently large to indicate multicollinearity [22]. The prediction performance of this model was evaluated through a leave-one-subject-out cross-validation (i.e., k-fold cross-validation with *k* = 18).

The values were considered significant at *p* < 0.05. All data were analyzed using JMP pro 16.0 (SAS Institute Inc., Cary, NC).

## Results

### Baseline characteristics

A total of 18 consecutive patients were included in this study, all of whom underwent EC-IC bypass (laterality of operation: 7 were right and 11 were left) without adverse events. Magnetic resonance angiography (MRA) confirmed bypass patency in patients 3–6 months after surgery. The basic characteristics are shown in Table 1. In all cases, the etiology was atherosclerosis, and the dominant hand was the right hand. There were no significant differences in age, sex, time scores, and accuracy scores between the left and right surgical sides.

### Visual cancellation task

**Fig. 1:**
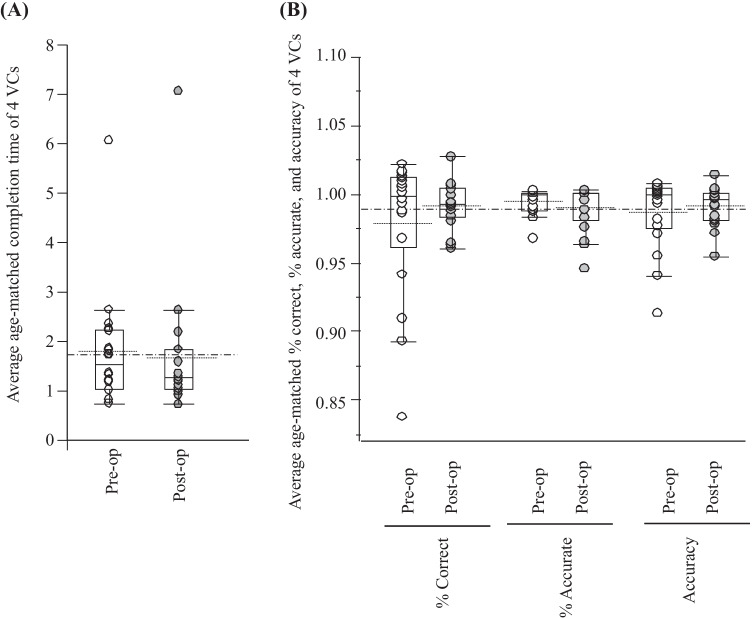
Box plots and dot plots of differences in the average age-matched completion time (**A**) and % correct answers, % accurate answers, and the accuracy score (**B**) between pre- (white) and post- (gray) extracranial-intracranial (EC-IC) bypass of the 4 visual cancellation (VC) subtests. Box plots show medians, quartiles, and 10th and 90th percentiles. The dotted line shows the average. Dash-dot-dash line shows the average pre- and post-operative completion time or % correct, % accurate, and accuracy score of 4 VC subtests. Score = 1 indicates the age-matched average

The time score (average of age-matched completion time for all four VC tasks) before EC-IC bypass was widely distributed in the range greater than 1, suggesting many cases to be slower than the average completion time (Fig. 1A). The median time score tended to be closer to 1 after EC-IC bypass (completion time became faster), but no significant difference was observed (Fig. 1A).

Compared with the age-matched completion time, the average age-matched % correct and % accurate answer of 4 subtests of VC and the accuracy score before EC-IC bypass tended to be distributed in a narrow range close to 1, and there was no remarkable change after extracranial-intracranial bypass (Fig. 1B, supplemental Table 1).

Therefore, the speed of the visual cancellation task tended to be slower, whereas accuracy was maintained before surgery.

### CVR change after extracranial-intracranial bypass

**Fig. 2:**
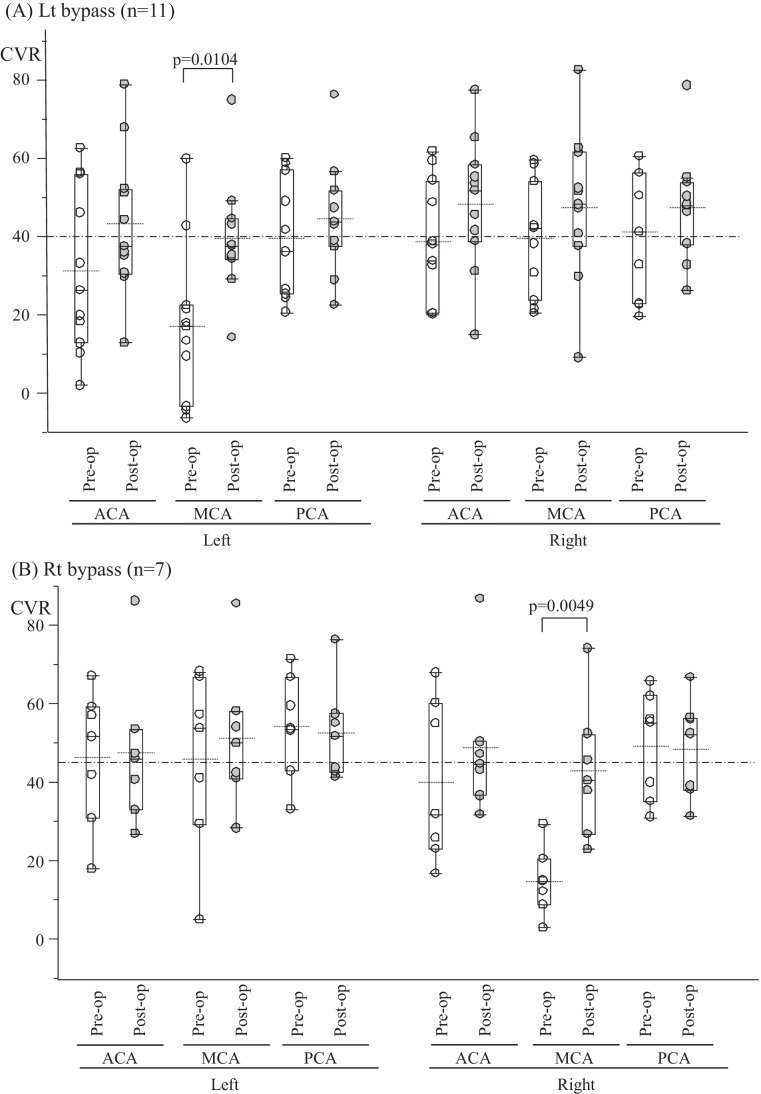
Box plots and dot plots of differences in CVR change in right or left ACA, MCA, and PCA territories between pre- (white) and post- (gray) EC-IC bypass (vertical lines indicate standard deviation) classified by treatment side (**A**; left, **B**; right). Box plots show medians, quartiles, and 10th and 90th percentiles. The dotted line shows the average. Dash-dot-dash line shows the average pre- and post-operative CVR. The *p* value was obtained using the Mann–Whitney *U* test

Figure 2 shows the CVR in bilateral ACA, MCA, and PCA territories before and after left (A) and right side (B) EC-IC bypass. Post-operative CVR in the MCA area on the surgical side was significantly higher than before surgery. The ACA and PCA areas also showed increased CVR after surgery, but not significantly. The EC-IC bypass in this study was considered to have improved CVR mainly in the cerebral hemisphere on the surgical side.

### Bivariate analysis

Spearman’s rank correlation coefficients between the time or accuracy score and CVR changes of 62 brain regions were examined on each surgical side. The correlations and *p*-values of all 62 brain regions are shown in supplemental Table 1 and summarized in Table 2.

**Table 2:** Summary of the relationship between change in the accuracy or time score and each regional CVR change

	Change in accuracy score		Change in time score	
	Lt EC-IC bypass	Rt EC-IC bypass	Lt EC-IC bypass	Rt EC-IC bypass
Number (%) with negative *ρ* (negative correlation)	4 (6.5%)	2 (3.2%)	49 (79.0%)	6 (9.7%)
Number (%) with positive *ρ* (positive correlation)	58 (93.5%)	60 (96.8%)	13 (21.0%)	56 (90.3%)
Number of *p*-value less than 0.1	10 (16.1%)	13 (21.0%)	1 (1.6%)	19 (30.6%)
Number of *p*-value of 0.05 or less	5 (8.1%)	6 (9.7%)	1 (1.6%)	9 (14.5%)

A summary of bivariate analyses of the time or accuracy scores and CVR changes in each brain region (62 brain regions in total) is shown. The number (% of 62 total brain regions) of Spearman’s rank correlation (*ρ*) values that are positive (or negative) and the number (% of 62 total brain regions) that have *p*-values less than 0.1 (or 0.05 or less) are shown separately on the surgical side. See the supplementary Table for all bivariate results

**Fig. 3:**
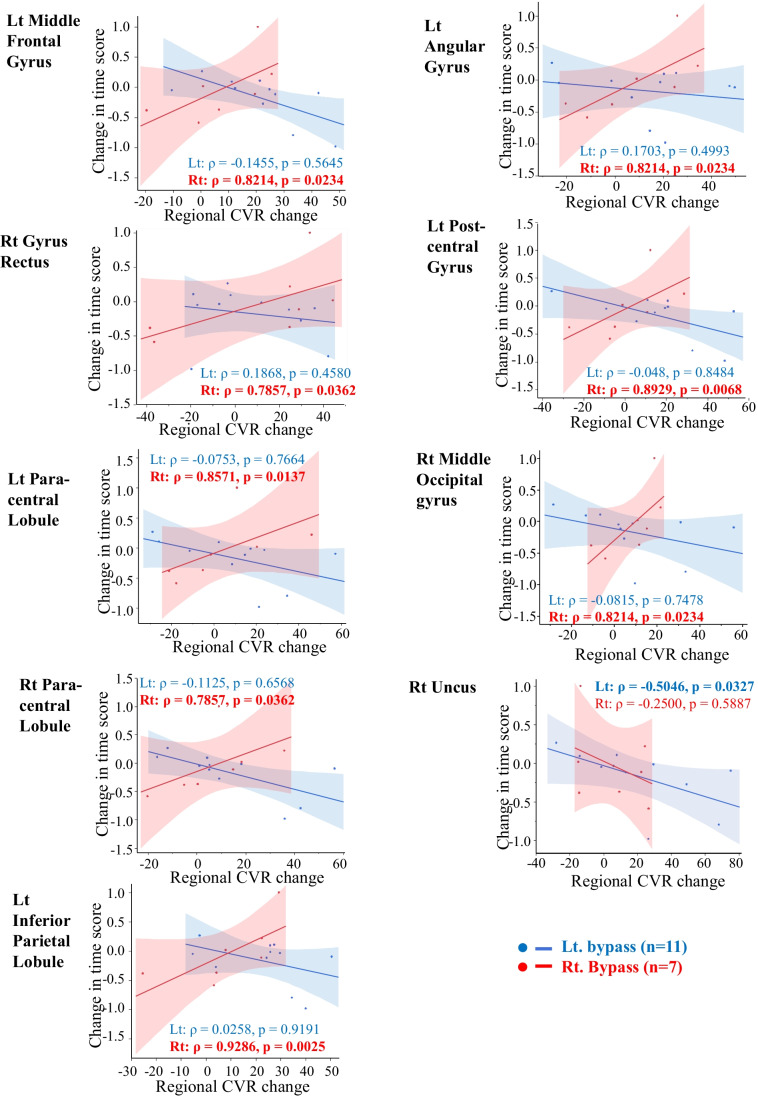
Scatter plots show the correlation between each regional CVR change and the time score change between pre- and post-EC-IC bypass. The results showing a *p*-value of 0.05 or less on bivariate analysis are shown (see supplemental Table 1 for all results). The solid lines represent simple linear regression lines. The shaded area indicates the 95% confidence interval of each regression line. The Spearman rank correlation coefficient (*ρ*) and *p* values are presented (bold when the *p* value is 0.05 or less). All the above are color-coded on the surgery side (blue indicating left and red indicating right EC-IC bypass)

The correlation between the time score and the CVR change in each brain region was negative on the left surgery side and positive on the right surgery side. The brain region showing a significant CVR change tended to be biased toward the right surgery side (Table 2, Fig. 3).

**Fig. 4:**
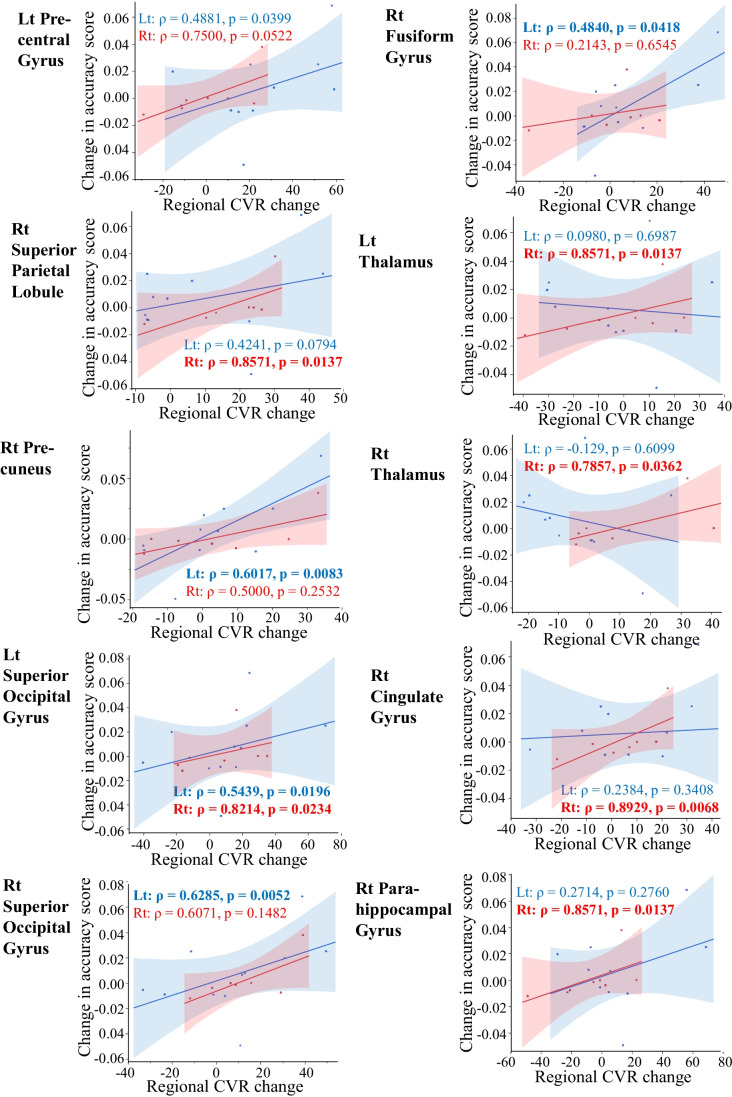
Scatter plots show the correlation between each regional CVR change and the accuracy score change between pre- and post-EC-IC bypass. Results showing *p*-values of 0.05 or less on bivariate analysis are shown (see supplemental Table 1 for all results). The solid lines represent simple linear regression lines. The shaded area indicates the 95% confidence interval of each regression line. The Spearman rank correlation coefficient (*ρ*) and *p* values are presented (bold when the *p* value is 0.05 or less). All the above are color-coded on the surgery side (blue indicating left and red indicating right EC-IC bypass)

On the other hand, the correlation between the accuracy score and the CVR change in each brain region was positive on both surgery sides. Moreover, the brain region showing a significant CVR change tended to be similar (Table 2, Fig. 4).

### Stepwise multiple regression analysis

Stepwise multiple linear regression analysis was performed to assess the relationship between the time score or accuracy score and CVR change in 10 or 11 brain regions with the *p*-value of 0.05 or less on the above bivariate analysis.

For the brain regions associated with the time score, a stepwise multiple regression model showed a low predicted value (adjusted *R*^2^ value = 0.1391), with 5 related regions remaining (Table 3).

**Table 3:** Stepwise multiple linear regression analysis predicting time score change

	Estimate	Std. error	*t* value	Pr( >|t|)	CI	Standard *β*	Logworth	VIF
(Intercept)	1.2425177	0.660593	1.88	0.0894	− 0.229375 to 2.71441	0		
CVR change in Rt uncus	− 0.016778	0.003943	− 4.25	0.0017	− 0.025565 to − 0.007992	− 1.10135	2.775	2.839741
CVR change in Rt middle occipital gyrus	0.0274737	0.009141	3.01	0.0132	0.0071053 to 0.047842	1.222319	1.879	7.0103066
CVR change in Rt paracentral lobule	− 0.01637	0.006567	− 2.49	0.0318	− 0.031001 to − 0.001738	− 0.77574	1.497	4.1043386
CVR change in Lt angular gyrus	0.007726	0.003939	1.96	0.0782	− 0.00105 to 0.0165021	0.39835	1.107	1.7479002
Age	− 0.017928	0.009745	− 1.84	0.0957	− 0.039641 to 0.0037857	− 0.40824	1.019	2.0870254
Sex [F]	0.114909	0.081888	1.4	0.1908	− 0.067549 to 0.297367	0.242789	0.0719	1.2687193
CVR change in Rt gyrus rectus	0.0008638	0.004467	0.19	0.8505	− 0.009089 to 0.0108163	0.053486	0.07	3.2417151

Variables entered into the model were age, sex, laterality of EC-IC bypass, and CVR change in Lt middle frontal gyrus, Rt gyrus rectus, Lt paracentral lobule, Rt paracentral lobule, Lt inferior parietal lobule, Lt angular gyrus, Lt postcentral gyrus, Rt middle occipital gyrus, Lt anterior cingulate, and Rt uncus. The table shows the final step of the analysis. The adjusted coefficient of determination (*R*^2^) calculated by leave-one-subject-out cross-validation was 0.1391. The VIF of each individual variable was < 3.5, indicating minimal collinearity

*β* standard partial regression coefficient, *CI* 95% confidence interval for each regression coefficient, *VIF* variance inflation factor

For the brain regions associated with the accuracy score, a stepwise multiple regression model showed a low predicted value (adjusted *R*^2^ value = 0.2533), with 4 related regions remaining (Table 4).

**Table 4:** Stepwise multiple linear regression analysis predicting accuracy score change

	Estimate	Std. error	*t* value	Pr( >|t|)	CI	Standard *β*	Logworth	VIF
(Intercept)	− 0.003299	0.004766	− 0.69	0.5009	− 0.013594 to 0.0069962	0		
CVR change in Rt cingulate gyrus	− 0.000876	0.000323	− 2.71	0.0177	− 0.001573 to − 0.000179	− 0.68727	1.752	2.4578475
CVR change in Rt precuneus	0.0008368	0.000345	2.43	0.0304	0.000092254 to 0.0015813	0.554067	1.517	1.9969942
CVR change in Lt precentral gyrus	0.0004376	0.000221	1.98	0.0698	− 0.00004089 to 0.0009161	0.444929	1.156	1.9448464
CVR change in Rt superior occipital gyrus	0.0004706	0.000256	1.84	0.0893	− 0.00008316 to 0.0010243	0.421314	1.049	2.019667

Variables entered into the model were age, sex, laterality of EC-IC bypass, and CVR change in Lt precentral gyrus, Rt superior parietal lobule, Rt precuneus, bilateral superior occipital gyrus, Rt fusiform gyrus, bilateral thalamus, Rt cingulate gyrus, Rt parahippocampal gyrus, and Rt uncus. The table shows the final step of the analysis. Adjusted coefficient of determination (*R*^2^) calculated by leave-one-subject-out cross-validation was 0.2533. The VIF of each individual variable was < 2.5, indicating minimal collinearity

*β* standard partial regression coefficient, *CI* 95% confidence interval for each regression coefficient, *VIF* variance inflation factor

## Discussion

In this study, the change in cognitive function after EC-IC bypass surgery was evaluated by dividing the results of VC into time and accuracy scores, and the relationships with the change in CVR were investigated for each brain region. Before EC-IC bypass, the speed of VC tended to be slower, whereas accuracy was maintained. The EC-IC bypass improved CVR mainly in the cerebral hemisphere (significantly in the MCA territory) on the surgical side. On bivariate analysis, when CVR increased post-operatively, accuracy improved on both the left and right surgical sides, but the time score was faster on the left but slower on the right surgical side. These results may indicate that speed and accuracy may be regulated separately in the left and right cerebral hemispheres when performing VC. This relationship between the time score and the accuracy score has been called the “speed-accuracy trade-off” in the research field of human kinematics (kinesiology) [18].

In the many previous studies of the “speed-accuracy trade-off,” inhibition of ongoing thought and action was focused when a mistake was noticed: if the stopping process wins, thought and action are inhibited (slow but more accurate); if the ongoing process wins, thought and action are executed (fast but less accurate) [23]. This inhibition mechanism has been thought to be related to the cortical-basal ganglia circuit; the basal ganglia output an inhibitory signal on the thalamus like a brake and control the speed and accuracy of some action initiated by cerebral cortical activity [24–27]. Moreover, the network systems coordinating motor control and visual signals were thought to be associated with some specific cortical regions, i.e., inferior parietal systems and the temporal lobe [28]. From the theory of the “speed-accuracy trade-off,” improvement of CVR after EC-IC bypass may directly increase accuracy or slow down processing speed and indirectly increase accuracy. The mismatch in the relationship between CVR changes and time scores in this study may indicate that the former is on the left and the latter is on the right operative side.

The present results suggest that the left and right cerebral hemispheres regulate speed and accuracy in a coordinated manner. Our previous study that examined the cognitive function related to car driving showed the negative impact of right hemisphere damage on the processing speed in stroke patients, suggesting the importance of preservation of the right hemisphere function for maintaining quality of life [16, 29]. Traditionally, in EC-IC bypass, greater attention has been paid to preservation of the left hemisphere than the right hemisphere. However, given the results of the present study, it is also necessary to consider whether the functions of both the left and right hemispheres are preserved.

### Study limitations

The number of subjects in the present study was small, and statistical analysis issues could have been a problem due to the large number of explanatory variables for the objective variable, the problem of multicollinearity, and overfitting.

Moreover, many previous papers examined the effect of EC-IC bypass on cognitive function with long-term follow-up (6 months to 2 years or more), but the present study had relatively short follow-up (3 months). In the future, it will be necessary to carry out analyses using data obtained from more cases with longer-term follow-up.

In the present study, CBF measurement was performed using IMP-SPECT and the graph plot method. This method is non-invasive but semi-quantitative, because it does not collect arterial blood [21]. However, the CVR calculated in this study is a method for measuring CBF at rest and after acetazolamide administration in 1 day and is thought to be suitable for comparison between patients in the same institution. Therefore, CVR was used for analysis instead of CBF. To clarify the relationship between cerebral hemodynamics and cognitive function, quantitative CBF measurement using arterial blood sampling or PET measurement will be needed.

The spatial resolution of the CVR images was low, and it was difficult to analyze the small anatomical structures. Thus, further study using a high-resolution method (i.e., MRI) will be needed to clarify the relationship between cognitive function and brain region.

Identification of dominant and non-dominant hemispheres is one of the key factors in brain imaging. In the present study, only hand dominance was examined. Therefore, it will be necessary in the future to identify dominant hemispheres using positron emission tomography (PET) or functional magnetic resonance imaging (fMRI).

The multivariate analysis in this study had low predicted values. The small number of cases may be the cause, but it is also considered that the correlation between the CVR change in each brain region and the time score showed an inverse correlation on the surgical side. In the future, it would appear to be necessary to increase the number of cases and consider non-linear multiple regression analysis, i.e., machine learning.

## Conclusions

This study suggested the following. In hemodynamic ischemia (both CBF and CVR reduced), processing speed might be adjusted so that accuracy would not be lowered by the speed-accuracy trade-off mechanism. More data will be needed before the clinical implications of these findings become clear. However, we have pointed out the possibility for the first time that it is necessary to pay attention to the “speed-accuracy trade-off” for each brain region when investigating the effect of EC-IC bypass on cognitive function. Therefore, considering the results of the present study, a different concept in the discussion of the relationship between cerebral hemodynamics and cognitive function is proposed. Future studies may use speed-accuracy imbalance as one of the diagnostic methods for stage 2 hemodynamic ischemia. In that case, it may be possible to consider surgical indications for bypass surgery in daily medical practice without special equipment.

## Supplementary Information

Below is the link to the electronic supplementary material.

Supplementary file1 (XLSX 16 KB)

## Data Availability

The datasets generated and/or analyzed in the current study are available from the corresponding author upon reasonable request.
